# The Quest for a Vaccine Against Coccidioidomycosis: A Neglected Disease of the Americas

**DOI:** 10.3390/jof2040034

**Published:** 2016-12-16

**Authors:** Theo N. Kirkland

**Affiliations:** Departments of Pathology and Medicine, University of California, San Diego, School of Medicine, San Diego, CA 92161, USA; tkirkland@ucsd.edu; Tel.: +1-858-459-7471

**Keywords:** *Coccidioides immitis*, *Coccidioides posadasii*, coccidioidomycosis, vaccine, immunology, fungi, T-cell mediated immunity

## Abstract

Coccidioidomycosis (Valley Fever) is a disease caused by inhalation of *Coccidioides* spp. This neglected disease has substantial public health impact despite its geographic restriction to desert areas of the southwestern U.S., Mexico, Central and South America. The incidence of this infection in California and Arizona has been increasing over the past fifteen years. Several large cities are within the endemic region in the U.S. Coccidioidomycosis accounts for 25,000 hospital admissions per year in California. While most cases of coccidioidomycosis resolve spontaneously, up to 40% are severe enough to require anti-fungal treatment, and a significant number disseminate beyond the lungs. Disseminated infection involving the meninges is fatal without appropriate treatment. Infection with *Coccidioides* spp. is protective against a second infection, so vaccination seems biologically plausible. This review of efforts to develop a vaccine against coccidioidomycosis focuses on vaccine approaches and the difficulties in identifying protein antigen/adjuvant combinations that protect in experimental mouse models. Although the quest for a vaccine is still in the early stage, scientific efforts for vaccine development may pave the way for future success.

## 1. Introduction

**Figure 1:**
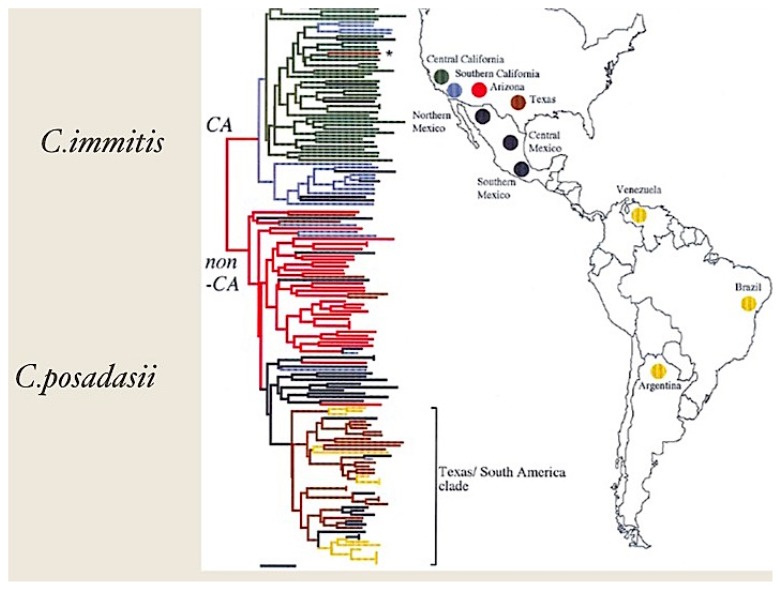
Geographic distribution and phylogeny of *C. immitis* and *C. posadasii*. This figure is adapted from reference [1]. CA refers to California (*C. immitis*) and non-CA refers to non-California (*C. posadasii*). The color coding is based on phylogenetic relatedness [1]. The publisher has granted the rights for reproduction of this figure.

*Coccidioides* spp. are one of the small number of primary pathogenic fungi capable of causing invasive disease in normal hosts. There are two sibling species of *Coccidioides*: *C. immitis* and *C. posadasii*. *C. posadasii* is found in southern Arizona, Texas and the arid regions of Mexico, Central and South America (Figure 1) [1]. Coccidioidomycosis is diagnosed much more commonly in the desert southwest region of the United States than in other desert regions of the Americas. Coccidioidomycosis to the west of the Tehachapi mountain range in the U.S. is usually due to *C. immitis*, while *C. posadasii* causes most of the infections east of that mountain range [1]. More than 75% of coccidioidomycosis in Mexico and South America are caused by *C. posadasii* [2]. As far as we can tell, the two sibling species do not cause different clinical syndromes, but few isolates are speciated. Travel to the desert increases the chances of infection but urban dwellers can become infected without such exposure. Disturbance of soil, for example with large construction projects or as happened with the Northridge earthquake in the Los Angeles region, increases the risk substantially [3,4].

Coccidioidomycosis is responsible for a great deal of morbidity and mortality in North America. *Coccidioides* spp. are normal soil flora in the desert regions of Southern California, as well as the heavily populated Phoenix region in Arizona [5]. A total of 25 million people, almost 10% of the population of the U.S., live in or adjacent to this endemic area. The number of cases of coccidioidomycosis has increased substantially between 2000 and 2011, coincident with a severe drought in California and Arizona [6]. One analysis of hospital admissions found that there were 25,000 coccidioidomycosis-associated admissions in California in the 2010–2011 period, with a cost of more than $2 billion United States Dollars (USD) [7]. A total of 30% of community acquired pneumonia in Tucson, Arizona, is due to *Coccidioides* spp. infection [8]. The annual rate of infection in 2011 was 250/100,000 in Arizona and 14/100,000 in California [9]. Coccidioidomycosis is a neglected disease. Despite the burden of coccidioidomycosis, the number of investigators studying this disease is very small and almost all pharmaceutical companies have little interest.

There are several excellent reviews of the epidemiologic, immunologic and clinical aspects of coccidioidomycosis [4,10,11,12]. Only a few pertinent aspects of the clinical disease will be discussed here. The majority of infections due to *Coccidioides* spp. will eventually resolve spontaneously, but the median duration of illness is four months [13]. About 3100 people/year die from coccidioidomycosis [9]. In some racial groups, such as Filipinos and African-Americans, the rate of dissemination is ten-fold higher than in the general population [14]. Pregnant women, the elderly and the immunosuppressed are also at a much higher risk for disseminated disease [14]. Once the infection disseminates, it is frequently a lifelong problem. Miliary dissemination and especially dissemination to the meninges are fatal when not treated appropriately. Therapy with amphotericin B is not uniformly effective and is often toxic. Less toxic therapy with azole drugs requires treatment for months to years and is expensive. Treatment of *Coccidioides* spp. meningitis appears to require lifelong therapy with currently available drugs [15]. For all these reasons, an effective vaccine for coccidioidomycosis is an important goal.

## 2. Microbiology

**Figure 2:**
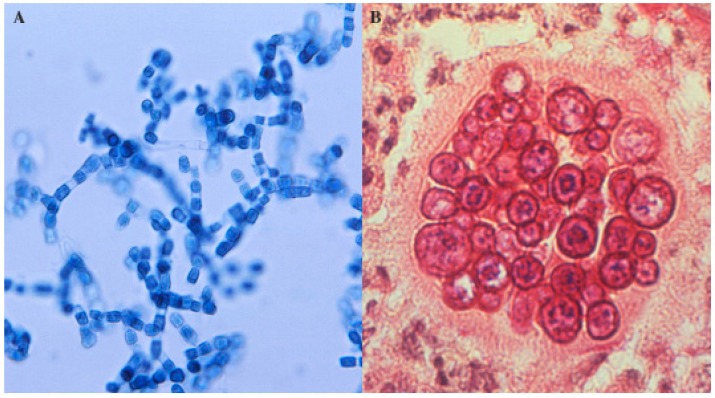
*Coccidioides* spp. arthroconidia and spherules. (**A**) Arthroconidia within hyphae (lacto-phenol cotton blue preparation) (**B**) Spherule containing endospores in tissue (periodic acid—Schiff stain). The images were obtained from the CDC (http://phil.cdc.gov/phil/details.asp). The images are in the public domain.

*Coccidioides* spp. is an unusual fungus because it can cause invasive disease in normal hosts. These organisms grow in a mycelial form in the soil at 25 °C and as a spherule within mammals at 37 °C. The mycelia form arthroconidia within their wall, every other cell degenerates and the arthroconidia are dispersed as the wind fractures the mycelia (Figure 2A). When inhaled by a susceptible host, they round up and form spherules, which divide to form internal endospores. When the spherule ruptures, the endospores escape and differentiate into spherules. A single spherule can contain hundreds of endospores (Figure 2B). 

There have been three studies of the global gene expression in mycelia and spherules by RNA-seq and microarray [16,17,18]. Between 7% and 20% of genes were differentially expressed in spherules compared to mycelia. Since almost all the organisms in human beings are in the spherule form, it seems appropriate to choose potential antigenic proteins that are expressed in spherules as prospective vaccines. It is not clear, however, whether proteins that are preferentially expressed in spherules compared to mycelia would be more effective vaccines.

## 3. Immunology

*Coccidioides* spp. elicit granuloma formation around spherules, a response associated with T-cell mediated immunity. A positive skin test to coccidioidin (an extract of hyphae) or spherulin (an extract of spherules) are a good prognostic signs [19]. These observations indicate that the immune response to coccidioidomycosis in human beings requires functional T-cells. Conversely, a high titer of complement-fixing antibody to *Coccidioides* is associated with a poor outcome [20]. The complement fixation antibody binds chitinase [21], so the anti-chitinase antibody does not play a protective role. The protective potential of antibodies specific for other antigens has not been investigated. 

Successful vaccine development requires an in vitro assay of human immunity so that the immunogenicity of the vaccine is documented and factors such as antigen preparation, formulation, dose, and adjuvants can be optimized. Determining the most useful assay has been difficult. Some of the methods used include the expression of the T-cell activation markers [22] or intracellular cytokines [23] comparing the response of lymphocytes from immune and control subjects to a mechanical spherule extract known as T27K. Immune cells produce more IL-2, TNF-α, IFN-γ and IL-17 in response to T27K than controls but the studies used complex extracts and had small numbers of subjects. There is strong evidence in mice that T-cells are required for immunity to coccidioidomycosis and that the predominance of Th1 to Th2 cells is important [10]. The importance of IL-17 producing T-cells for successful vaccination of mice with a live, attenuated mutant strain of *C. posadasii* has also been firmly established [24].

It is presumed that activated macrophages somehow kill or inhibit spherules but the mechanisms are completely unknown. Mice with a defect in their production of reactive oxygen intermediates are no more susceptible to experimental coccidioidomycosis than controls [25]. Inducible nitric oxide synthase also appears to play little, if any, role [26]. Resolving the mechanism(s) of protective immunity is important for understanding recovery from coccidioidomycosis.

## 4. Vaccination

### 4.1. Overview

Immunization against coccidioidomycosis seems feasible because second infections with *Coccidioides* spp. are extraordinarily rare. Ideally, a vaccine would prevent, or drastically reduce, the incidence of infection, but one would especially like to prevent symptomatic infection and to protect those at high risk for disseminated disease. The mechanisms of the genetically determined increased risk of disseminated disease in African-Americans and Filipinos are not known but an analogous genetically determined difference in susceptibility to infection occurs in inbred mouse strains [27]. Genetically susceptible mouse strains can be successfully vaccinated with a variety of *Coccidioides* spp. antigens which suggests that individuals who are genetically susceptible to disseminated infection may also be successfully immunized.

Because human infection almost always conferred life-long immunity, the search for an effective vaccine was initiated long before the era of modern immunology. The first decision about vaccination experiments is how protective vaccination is defined and which model is most realistic. Details of experimental intranasal infections in mice are a major variable from one laboratory to the next. These include the species and strain of *Coccidioides* spp., the mouse strain, the number of organisms in the challenge, the dose and timing of the antigen and the adjuvant used for immunization. Using BALB/c mice—the most susceptible strain for the experimental model—is a very stringent test of vaccine activity. C57BL/6 mice are a less demanding challenge model and one that may be more reasonable. It is not clear which experimental infection protocol correlates with protective immunity in human beings because less than ten arthroconidia are frequently fatal in BALB/c and C57BL/6 mice, whereas most human beings recover from natural infection spontaneously.

Table 1 shows many of the live attenuated mutants, biochemical preparations and recombinant protein antigens that have been tested. In the 1960s, it was found that immunizing mice with formalin-killed spherules protected them against subsequent intranasal infection [28]. This finding has been confirmed repeatedly, although enhanced innate immunity to glucan may play a large part. Spherule extracts, designated T27K, are also protective [29]. However, it is not possible to characterize these extracts since they contain glycoproteins, carbohydrates and lipids, which may affect the innate immune system. In addition, the techniques for culturing spherules, preparing the extracts, and achieving reproducible products are too cumbersome to be practical as human vaccines.

### 4.2. Live Attenuated Mutants

Several live attenuated mutant vaccines have been tested. One mutant developed in the 1960s by UV irradiation is axuotrophic, temperature sensitive and an effective vaccine [27]. The exact genetic mutations causing the attenuation are unknown. A more targeted approach was taken after the genome of *Coccidioides* spp. had been sequenced. One mutant was developed by disrupting two chitinase genes; the genetically engineered organism was unable to endosporulate [30]. Immunization with this living mutant was safe and very highly protective in mice. Very similar results were found with another mutant with deletion of the homolog of the *CPS1* gene, which codes for virulence in *Cochliobolus heterostrophus* [31]. These mutant strains are more protective vaccines than recombinant proteins. If vaccine efficacy were the only consideration, these mutants would be the most attractive candidates. However, given the fact that 60% of infected people spontaneously recover from coccidioidomycosis, it seems unlikely that human vaccination with a live attenuated mutant would be acceptable to the U.S. Food and Drug Administration (FDA) or the general public.

### 4.3. Vaccine Candidate Proteins

Many investigators have pursued recombinant protein vaccines. This was feasible because the genome sequence of both *C. immitis* and *C. posadasii* was available [39]. However, not all proteins expressed on the surface of the spherule are effective vaccines. For example, spherule outer wall glycoprotein is highly expressed on the spherule surface and is highly immunogenic but is not protective [40]. Dozens of other antigenic proteins are also not protective (unpublished). Unfortunately there is currently no way to predict which spherule proteins will elicit protective immunity.

Antigen 2 is a complex antigen extract defined by interaction with antisera in the 1970s [41]. The cDNA coding for one of these proteins was cloned by two groups and named antigen 2 [42] or proline rich antigen [43]. This protein came to be known as Ag2/PRA. Although Ag2/PRA is found below the surface of the spherule, it was found to be an effective protein and DNA vaccine in an intranasal challenge [43]. There have been at least several dozen experiments showing that Ag2/PRA vaccination is protective in C57BL/6 mice challenged with 10–50 arthroconidia [37,44]. Nevertheless, the effectiveness of Ag2/PRA as a vaccine has been somewhat controversial which may be due to methodological differences, such as size of inoculum and choice of mouse strain [10]. The difference in protein sequence in the two species is a potential problem and no studies have compared protection with both *Coccidioides* spp. However, Ag2/PRA is extremely conserved in a number of isolates of both species, so immunization with this protein would be expected to protect against *C. immitis* and *C. posadasii* [45]. Other vaccine candidate proteins are also highly conserved between the two species (unpublished).

A number of other recombinant proteins based on the genomic sequence of *C. posadasii* and *C. immitis* that was becoming available showed moderate activity as vaccines (see Table 1). One fruitful approach to find potential antigens was to look for proteins that were predicted to have signal peptides and GPI anchors, since those proteins would be predicted to be on the cell surface [36,46]. Another approach has been to look at proteins expressed by spherules using proteomics to choose which proteins to investigate [36,47]. The combination of two or three modestly effective proteins into vaccines resulted in much more effective vaccines than the single proteins [36,37]. For example, a domain of Ag2/PRA fused with the Coccidioides specific antigen yielded a recombinant protein that was more protective than either one alone [37]. The combination of α-mannosidase, phospholipase and aspartyl protease was a particularly effective vaccine, when given as three proteins or as predicted HLA binding epitopes within a single protein [46]. The multiple protein or epitope approach seemed to be the most promising subunit vaccine but, further studies of the combination antigen approach have not been published. More recently, it has been reported that calnexin, a highly conserved fungal protein that is expressed on the cell wall, is a modestly effective vaccine against a variety of different fungi including *Coccidioides* spp. [35]. Results of further development of these proteins into a clinically useful vaccine has not been published. This approach may lead to a useful vaccine for all dimorphic fungi and some opportunistic fungi, which would be a major step forward.

## 5. Other Issues

The type of adjuvant used with a subunit vaccine can be critical for protective capacity. Most of the recombinant vaccine studies were done with monophosphoryl lipid A and CpG or CpG alone as an adjuvant. Adjuvants are best understood in terms of the toll like receptor (TLR) they activate and more rational choices can now be made [48]. Comparisons of protein combinations to each other and comparisons of adjuvants would be needed to determine the most effective vaccine adjuvant combination.

The testing of potential vaccines in a larger animal, such as the macaque, is also important for pre-clinical development. An attempt to study vaccination with Ag2/PRA CSA fusion protein and intranasal challenge in Cynomologus macaques had equivocal results because the unimmunized macaques were not heavily infected, so any effect of vaccination was difficult to discern [49]. Only one experiment was done. Experimental infection in the macaque might be a very attractive model if an appropriate infectious challenge was established, and the protective ability of immunization with a live attenuated mutant was shown.

There has been no experimental work on therapeutic vaccines, even though those might be very attractive options for coccidioidomycosis, since they could be selectively used in patients with severe disease. However, therapeutic vaccines would need to be rapidly effective since the unvaccinated mice usually die within two to four weeks.

## 6. Human Trial

There has been one human trial of a coccidioidomycosis vaccine, conducted in the 1980s, using killed whole spherule immunization. A double-blind human study compared vaccine to placebo, using clinical outcomes as an endpoint [32]. That study, involving almost 3000 people, demonstrated that only a minority of the vaccinated people became skin-test positive to coccidioidin. There was no difference in the number of cases of coccidioidomycosis or the severity of the disease in the vaccinated group compared to the placebo control group, although the number of clinical cases was low. One explanation for the ineffectiveness of this vaccine may be that relatively small numbers of killed *Coccidioides* spherules could be injected into humans without unacceptable local side effects of pain and swelling. Clearly, toxicity in human beings, lack of good methods for measuring immunogenicity, and year-to-year variability in the incidence of infection are all potential problems for human vaccine trials. Identification of a test population that is not immune to *Coccidioides* spp. but is at high risk for developing the infection is another issue. Obtaining approval of the vaccine (including protein and adjuvant) by the FDA and the support of a pharmaceutical company would also be be necessary. Finally, convincing people to volunteer for a vaccine study would be essential. Although this list seems daunting, all vaccines must meet these requirements.

## 7. A Path to a Human Vaccine

**Figure 3:**
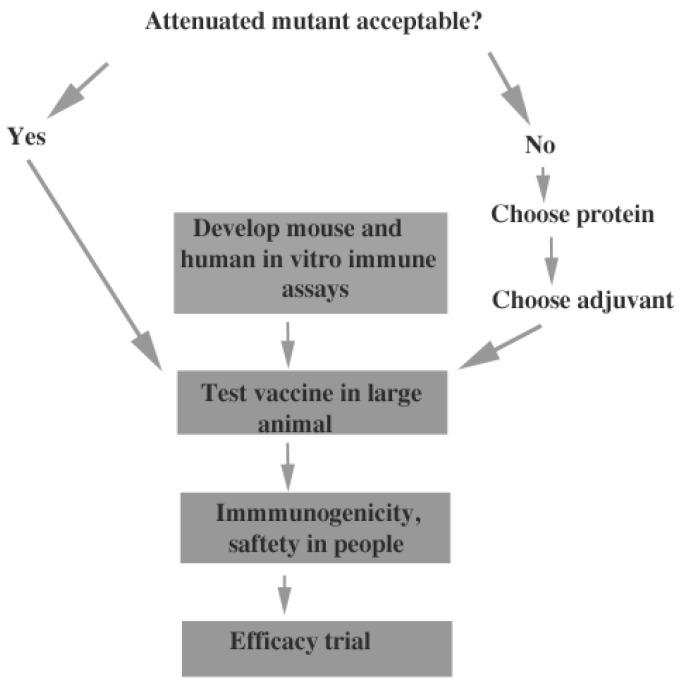
Flowchart for vaccine development.

Figure 3 shows some of the tasks required to reach a human vaccine. The issue of acceptability of live, attenuated mutants as vaccines needs to be addressed by discussions with the FDA and pharmaceutical companies. A live attenuated vaccine would probably be more protective but much less acceptable than a subunit vaccine, particularly for an infection that is frequently asymptomatic. Perhaps development of a live attenuated vaccine for veterinary use would be a useful incremental approach, since some species of pet animals are susceptible to infection (farm animals can also be infected but do not develop serious disease) [50]. If acceptance of vaccination with live mutants seems unlikely, more developmental studies of recombinant protein vaccines and protein/adjuvant combinations need to be done. This would require comparison of candidate proteins, or combinations of proteins, with the same adjuvant in mice, and testing of the most promising protein or combination of proteins, with a variety of adjuvants in mice. Once vaccine efficacy was established in mice, further testing in larger animals would be important. Non-human primates would be the best candidates but testing the vaccine in any animal other than mice would be useful. Regardless of the type of vaccine to be tested, validated immunologic tests in mice, larger animals and humans must be developed to ensure that vaccination elicits a robust immune response. One approach to this issue would be to compare the response to immunization with antigens that are protective to those that are not protective. Finally, testing of toxicity and immunogenicity in humans before an efficacy trial would be critical. No vaccine candidate, other than formalin-killed spherules, has been tested in humans because the vaccine work that has been done is at an early stage and these requirements have not been met.

Careful and realistic planning of an efficacy trial will be essential. A non-immune population with a high risk for infection must be identified. An endpoint involving disseminated disease is probably unrealistic because it would require a very large human trial since dissemination is relatively uncommon. Prevention of infection would be a difficult endpoint because half of the infections are asymptomatic. Decreasing symptomatic infection as an endpoint would require quantitative methods for measuring disease severity. Early involvement of a pharmaceutical company is critical; factors such as patents, cost of production, formulation and many other issues influence the decision to develop a vaccine. Pharmaceutical companies have shown little interest to date, probably because the vaccine experiments are at an early stage and the disease is geographically limited. Development of a vaccine for coccidioidomycosis will be a major challenge but a vaccine would be a great advance in prevention of this disease and reduction of the symptomatic illnesses that it causes. Lessons from this effort might be applicable to vaccine development for other pathogenic fungi as well.

## Figures and Tables

**Table 1:** Vaccine antigens.

Antigen	Form	Adjuvant	Activity	Reference
Live attenuated mutants	N/A ^a^	N/A	Active	[30,31]
Formalin-killed spherules	N/A	N/A	Active in mice but not humans	[28,32]
Spherule extract	N/A	Various	Active	[29]
Ag2/PRA	Protein, DNA	Various	Moderately active Inactive	[33,10]
β-glucanosyltransferase	Protein	CpG-ODN ^b^	Moderately active	[34]
Calnexin	Protein	Glucan and Adjuplex	Modestly active	[35]
Aspartyl protease	Protein	CpG-ODN	Moderately active	[36]
CSA ^c^	Protein	CpG-ODN and MPLA ^d^	Modestly active	[37]
Ag2/PRA and CSA fusion protein	Protein	CpG-ODN and MPLA	Highly active	[37]
Phospholipase, α-mannosidase and aspartyl protease	Protein	CpG-ODN	Highly active	[36,38]

^a^ Not applicable; ^b^ cytosine triphosphate deoxynucleotide—guanine triphosphate deoxynucleotide immunostimulatory polymer; ^c^ Coccidioides specific antigen; ^d^ monophosphoryl lipid A.
